# A highly efficient method for the production and purification of recombinant human CXCL8

**DOI:** 10.1371/journal.pone.0258270

**Published:** 2021-10-15

**Authors:** Sophie McKenna, Sean Patrick Giblin, Rosemarie Anne Bunn, Yingqi Xu, Stephen John Matthews, James Edward Pease

**Affiliations:** 1 Department of Life Sciences, Imperial College London, London, United Kingdom; 2 National Heart and Lung Institute, Imperial College London, London, United Kingdom; Katholieke Universiteit Leuven Rega Institute for Medical Research, BELGIUM

## Abstract

Chemokines play diverse and fundamental roles in the immune system and human disease, which has prompted their structural and functional characterisation. Production of recombinant chemokines that are folded and bioactive is vital to their study but is limited by the stringent requirements of a native N-terminus for receptor activation and correct disulphide bonding required to stabilise the chemokine fold. Even when expressed as fusion proteins, overexpression of chemokines in *E*. *coli* tends to result in the formation of inclusion bodies, generating the additional steps of solubilisation and refolding. Here we present a novel method for producing soluble chemokines in relatively large amounts via a simple two-step purification procedure with no requirements for refolding. CXCL8 produced by this method has the correct chemokine fold as determined by NMR spectroscopy and in chemotaxis assays was indistinguishable from commercially available chemokines. We believe that this protocol significantly streamlines the generation of recombinant chemokines.

## Introduction

Chemokines are key players in the recruitment and retention of leukocytes at tissue locations and function by activating specific G protein-coupled receptors (GPCRs) [1]. Over the last 30 years, chemokines have been the focus of much global research, since together with their receptors, they have been implicated in the pathogenesis of several clinically important diseases, ranging from atherosclerosis to HIV-infection [2]. Recombinant chemokine production has been key to these efforts but is not without challenges. Methods that focus on chemical synthesis or expression in *E*. *coli* typically yield unfolded, reduced, and inactive chemokines, necessitating refolding steps that can be time consuming and challenging to optimise. Generation of the native N-terminus of a chemokine, essential for activity, is a stringent requirement since truncation or extension can severely affect receptor activation. In mammalian hosts, a signal peptide of around 20 amino acids is typically cleaved to liberate the mature chemokine with full agonist activity. This process has to be mimicked in prokaryotic expression systems.

Pioneering studies by the groups of Ian Clark-Lewis and colleagues championed the use of solid-phase peptide sequencing. In particular, studies of the CXC chemokine CXCL8 (formerly known as interleukin-8) highlighted a requirement for the chemokine N-terminus for full agonist activity [3–5]. Expression of chemokines incorporating an N-terminal 6-Histidine tag such as those expressed in the pET23a vector [6] allows for simple purification by IMAC but the N-terminal extension generally leads to a loss of bio-activity. A classic example of this is the molecule Met-RANTES, a recombinant form of the chemokine CCL5 produced by Amanda Proudfoot and colleagues, which by virtue of the retained N-terminal methionine residue was serendipitously found to be a potent antagonist of multiple chemokine receptors, including the HIV-1 receptor CCR5 [7, 8]. Thioredoxin fusion has been utilised to promote disulphide bonding and generate the native chemokine N-terminus through Factor Xa mediated cleavage [9–12]. However, application of the thioredoxin fusion more widely is limited by the promiscuity of Factor Xa, which is prone to cutting at cryptic secondary sites in the protein of interest [13].

Recent advances, such as those published by the groups of Brian Volkman and Tracey Handel, have utilised *E*. *coli* expression systems in which an N-terminal SUMO partner is fused to the mature chemokine sequence [14, 15]. The SUMO partner can be readily and specifically cleaved by ubiquitin like protease-1 (ULP1), if an SMT3 cleavage site is incorporated upstream of the native N-terminal sequence. However, despite this advance, overexpression of SUMO-chemokine fusion proteins typically results in their appearance as insoluble aggregates within *E*. *coli* inclusion bodies, requiring a subsequent refolding step that can be inefficient and lead to reduced yields.

Interleukin-8 (IL-8 or CXCL8) is a member of the ELR+ CXC chemokine family and has been studied extensively, [16–19]. CXCL8 acts as a major recruitment factor for neutrophils expressing the chemokine receptors CXCR1 and CXCR2 on their surface [20] Interestingly, CXCL8 has been reported to undergo post-translational modification, notably N-terminal truncation by endogenous peptidases, which can markedly affect the potency and efficacy at both CXCR1 and CXCR2 reviewed by Vanheule and colleagues [21]. The 72 amino acid variant of CXCL8, CXCL8 (6–77), has been most extensively studied and is the predominant form isolated by activated PBMCs [22].

CXCL8 exists dynamically as a monomer or homodimer at low and high concentrations, respectively, with distinct recruitment profiles observed [23]. The high-resolution structure of CXCL8 has been determined by solution NMR and X-ray crystallography with the concentrations required for these studies associated with CXCL8 dimerization [24, 25]. The structure of the monomer subunit within the dimer encompasses an N-terminal loop followed by three antiparallel β-strands, and a C-terminal α-helix. Mutations that trap the monomeric or dimeric state have been structurally characterised and are consistent with the earlier studies, showing that the monomeric form differs with some disorder observed in the C-terminal helix [26].

Here we describe an optimised protocol for the purification of chemokines, using CXCL8 (6–77) as an exemplar, without additional refolding requirements. A recombinant SUMO-CXCL8 fusion protein was purified to homogeneity from the soluble lysate of *E*. *coli* Shuffle T7 Express cells via IMAC (immobilized metal affinity chromatography). Cleavage of the SUMO tag was efficient, and a second IMAC step resulted in the purification of CXCL8 with a native N-terminus. The chemokine produced by this protocol was shown to be correctly folded by NMR spectroscopy with bio-activity indistinguishable from that of commercially available CXCL8 as demonstrated by chemotaxis assays. This method could be applicable to wider chemokine production efforts, with the rapidity of this protocol (expression and purification of the construct takes 5 days) a distinct advantage over traditional recombinant refolding approaches.

## Materials and methods

### Materials

All materials were purchased from Sigma-Aldrich (Poole, UK) unless otherwise stated. Recombinant CXCL8 (6–77) and a human IL-8/CXCL8 DuoSet ELISA kit were purchased from BioTechne (Abingdon, UK). The pCI-CXCR1 construct was a kind gift of GlaxoSmithKline (Stevenage, UK). Oligonucleotide production and Sanger sequencing services were provided by MWG-Biotech (Ebersberg, Germany).

### Expression and purification of ULP1

*Saccharomyces cerevisiae* Ubiquitin-like-specific protease 1 (ULP1) was expressed in pET-28a, a gift from Dr Zhihao Wang and Dr Bing Liu. The construct covers residues 403 to 621 and is in-frame with the N-terminal His-tag and thrombin cleavage site in the vector. ULP1 was expressed in BL21(DE3) cells grown at 37°C until an optical density at 600 nm of 0.6 to 0.8, induced with 1 mM IPTG for 4 hours, and harvested by centrifugation (5000 x g for 15 minutes). Pellets were resuspended in 20 mM Tris.HCl pH 8.0, 350 mM NaCl, 20 mM imidazole, 5% glycerol, 1mM β-mercaptoethanol and sonicated. Cell lysate was clarified at 38000 x g for 45 minutes and loaded on a 5ml HisTrap FF crude column (GE Healthcare) pre-equilibrated with lysis buffer. The column was washed with 10 column volumes of lysis buffer and ULP1 was eluted in lysis buffer with 300 mM imidazole. The eluate was applied to an S75 16/600 (GE Healthcare) size exclusion column equilibrated with 20 mM Tris.HCl, 150 mM NaCl, 1 mM Dithiothreitol (DTT) for further purification.

### Generation, expression and purification of CXCL8

The pUC19 CXCL8 construct was a gift from Dr Joost Openheim (Addgene plasmid #17610), [27]. The cDNA sequence encoding the mature 72 amino acid chemokine (6–77) was amplified by the primers SUMO3-CXCL8 Forward: 5’-CAG CAG CAG ACG GGA GGT AGT GCT AAA GAA CTT AGA TGT CAG-3’ and SUMO3-CXCL8 Reverse: 5’-CTC GAA TTC GGA TCC TCA TGA ATT CTC AGC CCT CTT CAA AAA-3’ using CloneAmp^TM^ HiFi Premix (Takara Bio Europe SAS, Saint-Germain-en-Laye, France) according to the manufacturer’s instructions. Using the same premix, the pSUMO-3 vector (Lifesensors Inc, Malvern, USA) was linearized using the primers SUMO3 Forward: 5’-TGA GGA TCC GAA TTC GAG CTC-3’ and SUMO3 Reverse 5’-ACC TCC CGT CTG CTG CTG G-3’. PCR products underwent *DpnI* fast digestion (ThermoFisher Scientific, Paisley, UK) prior to ligation using the In-Fusion HD system (Takara). The authenticity of the pSUMO-3-CXCL8 open reading frame was verified by Sanger sequencing on both strands of the plasmid (MWG Eurofins, Ebersberg Germany).

The pSUMO3-CXCL8 plasmid was subsequently introduced into chemically competent Shuffle T7 Express *E coli* (New England Biolabs, Hitchin, UK) and following selection, colonies were grown in LB media or minimal medium containing ^15^NH_4_Cl a as the sole nitrogen source, both supplemented with carbenicillin. Cells were grown at 30°C until an optical density at 600 nm of 0.6 to 0.8 was reached, then protein production induced with 1 mM isopropyl-β-D-thiogalactoside (IPTG), grown for 4 hours at 30°C, and finally harvested by centrifugation at 5000 x g for 15 minutes.

Bacterial pellets were resuspended in buffer A (20 mM Na phosphate pH 6.7, 500 mM NaCl, 20 mM imidazole) supplemented with a cOmplete^TM^ Mini, EDTA-free Protease Inhibitor tablet (Roche, Welyn Garden City, UK) and sonicated. Cell lysates were clarified by centrifugation at 38000 x g for 45 minutes and loaded onto a 5ml HisTrap FF crude column (Cytiva, Amersham, UK) pre-equilibrated with buffer A. The column was washed with 5 column volumes of buffer A followed by a gradient over 20 column volumes to 100% buffer B (20 mM Na phosphate pH 6.7, 500 mM NaCl, 500 mM imidazole). The eluate was dialysed into 20 mM Na phosphate pH 6.7, 150 mM NaCl, 10% glycerol overnight at 4°C overnight. ULP1 was dialysed into the digestion buffer concurrently but separately. SUMO-3 removal was performed by incubating ULP1:SUMO3-CXCL8 at a ratio of 1:25 at 4⁰C for 48 hours with agitation. Separation of cleaved CXCL8 from the SUMO-3 tag and ULP-1 was achieved by loading the digested sample onto a 5ml HisTrap FF crude column pre-equilibrated with buffer A and eluting with buffer A. ULP1, undigested SUMO3-CXCL8 and SUMO3 tag were subsequently eluted from the column with buffer B.

### NMR spectroscopy

1D ^1^H spectra were recorded on 100 μM CXCL8 in 20 mM Na phosphate pH 6.7, 150 mM NaCl at 298 K. Spectral assignment of [*U*-^15^N]-labelled CXCL8 was performed at 500 μM in 20 mM Na phosphate pH 6.5, 100 mM NaCl, 10% D_2_O acquired at 298 K. ^15^N-HSQC, ^15^N-HSQC-TOCSY and ^15^N-HSQC-NOESY spectra were recorded and used to obtain the ^1^H and ^15^N assignments [28–30]. Spectra were processed with NMRPipe [31] and analysed using CCPN Analysis version 2.4 [32]. All spectra were recorded on a Bruker Avance III HD 800 MHz spectrometer equipped with a triple-resonance cryoprobe.

### SEC-MALS

All experiments were conducted at room temperature on a system comprising a Wyatt miniDAWN TREOS multi-angle light scattering detector and a Wyatt Optilab T-rEX refractive index detector coupled with a Superdex 75 10/300 GL (G.E. Healthcare). CXCL8 was analysed at 4.2 mg/ml (0.5 mM) in 20 mM Na phosphate pH 6.5, 100 mM NaCl.

### Determination of CXCL8 reactivity and concentration by ELISA

The Human CXCL8 DuoSet ELISA kit (Bio-Techne, Abingdon, UK) was used to determine both antibody recognition and the accurate concentration of CXCL8 produced in the laboratory. The signal detected from diluted CXCL8 samples was compared to commercially available CXCL8 standards generated by serial dilution from 0.015–2 ng/mL.

### Culture and transfection of pre-B cells

The L1.2 pre-B cell line was cultured as previously described in "complete" RPMI consisting of base RPMI 1640 medium with Glutamax-I, supplemented with 25 mM HEPES, 10% fetal calf serum, 1 mM sodium pyruvate, 1 mM 2-mercaptoethanol, 1 x non-essential amino acids and 1 x penicillin/streptomycin [33]. Cells were maintained at a density below 1.5x10^6^ cells/mL. Transient transfection of L1.2 cells with either the pCI-CXCR1 or pCDNA3-CXCR2 constructs was carried out as previously described using electroporation [34]. Briefly, 1x10^7^ L1.2 cells were incubated with 10μg of plasmid DNA in 800μl of base RPMI media. Electroporation was with carried out in a 0.4cm BTX cuvette (VWR, Lutterworth, UK) at 330V and 975μF. Cells were allowed to recover for twenty minutes prior to seeding at 1x10^6^ cells/mL in complete RPMI supplemented with 10mM sodium butyrate. Cells were used for flow cytometry and chemotaxis assays 24 h following transfection.

### Flow cytometry

Transfected cells were stained with either a PE-conjugated anti-human CXCR1 mAb (BioTechne), or a APC-conjugated anti-human CXCR2 mAb (Biolegend) or relevant conjugated isotype controls as previously described [34]. Cells were analysed on a FACSCalibur (BD Biosciences, Wokingham, UK) part of the Sir Alexander Fleming Building Flow Cytometry Facility at Imperial College London. Exclusion of TOPRO-3 and/or forward/side scatted profiles were used to distinguish live cells from dead cells or cell fragments.

### Chemotaxis assays

Chemotaxis assays were carried out using a modified Boyden chamber as previously described in detail [34]. Briefly, serial dilutions of recombinant CXCL8 (6–77) were generated using chemotaxis buffer (RPMI + 0.1% BSA) and plated in duplicate in the lower wells of a Chemo TX 101–5 chemotaxis plate (Neuroprobe, MD). The permeable membrane was placed on top and CXCR1 or CXCR2 transfectants were resuspended to a concentration of 1x10^7^ cells/mL in chemotaxis buffer of which 20μl were placed on the membrane above the relevant wells. The plate was placed in a humidified chamber at 37°C, 5% CO_2_. Cells were allowed to migrate for 5 hours after which the numbers of migrating cells were enumerated as previously described using the dye Cell Titer Glo (Promega, Southampton UK) [35]. Data were normalised to the signal generated from 2x10^5^ cells (input) and expressed as the percentage of cells migrated.

## Results

### CXCL8 expression and purification

**Fig 1:**
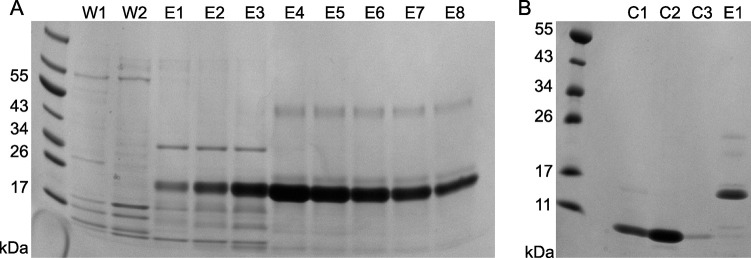
Purification of CXCL8. (A) SDS-PAGE analysis of CXCL8-SUMO3 nickel affinity purification. The lanes are labelled wash (W1 and W2) and elution (E1-E8). (B) SDS-PAGE gel analysis of nickel affinity purification of CXCL8 post SUMO3 tag digestion. The lanes are labelled CXCL8 elution (C1-C3), and elution (E1). CXCL8 is separated in the wash step from tagged ULP1, undigested CXCL8-SUMO3 and the SUMO3 tag. The molecular weights are as follows; CXCL8-SUMO3–20 kDa, CXCL8–8.4 kDa and SUMO3–11.7 kDa.

Shuffle T7 Express *E*. *coli* facilitates the formation of disulphide bonds in the cytoplasm, typically yielding more of the desired product than periplasmic expression. This strain was utilised to overexpress the SUMO-CXCL8 fusion protein, producing a significant quantity of soluble protein (S1 Fig). Post sonication and lysate clarification, a single nickel affinity purification step yielded pure SUMO-CXCL8 for tag digestion (Fig 1A). Fractions were pooled following SDS-PAGE analysis and dialysed. ULP1 was dialysed separately into reducing agent free buffer to ensure CXCL8 would not be reduced upon digestion. Although ULP1 is a cysteine protease, efficient cleavage has been observed in the absence of β-mercaptoethanol [36]. However, complete removal of the SUMO-3 tag in the absence of a reducing agent requires a higher ULP1 concentration than under standard buffer conditions. Tag removal was therefore carried out at a ratio of 1:25 (ULP1:SUMO fusion substrate) and incubated at 4°C overnight. Re-purification of the sample using IMAC facilitated the separation of cleaved, untagged CXCL8 from the other tagged components in a single affinity step (Fig 1B).

### CXCL8 produced in-house is chemotactically active

**Fig 2:**
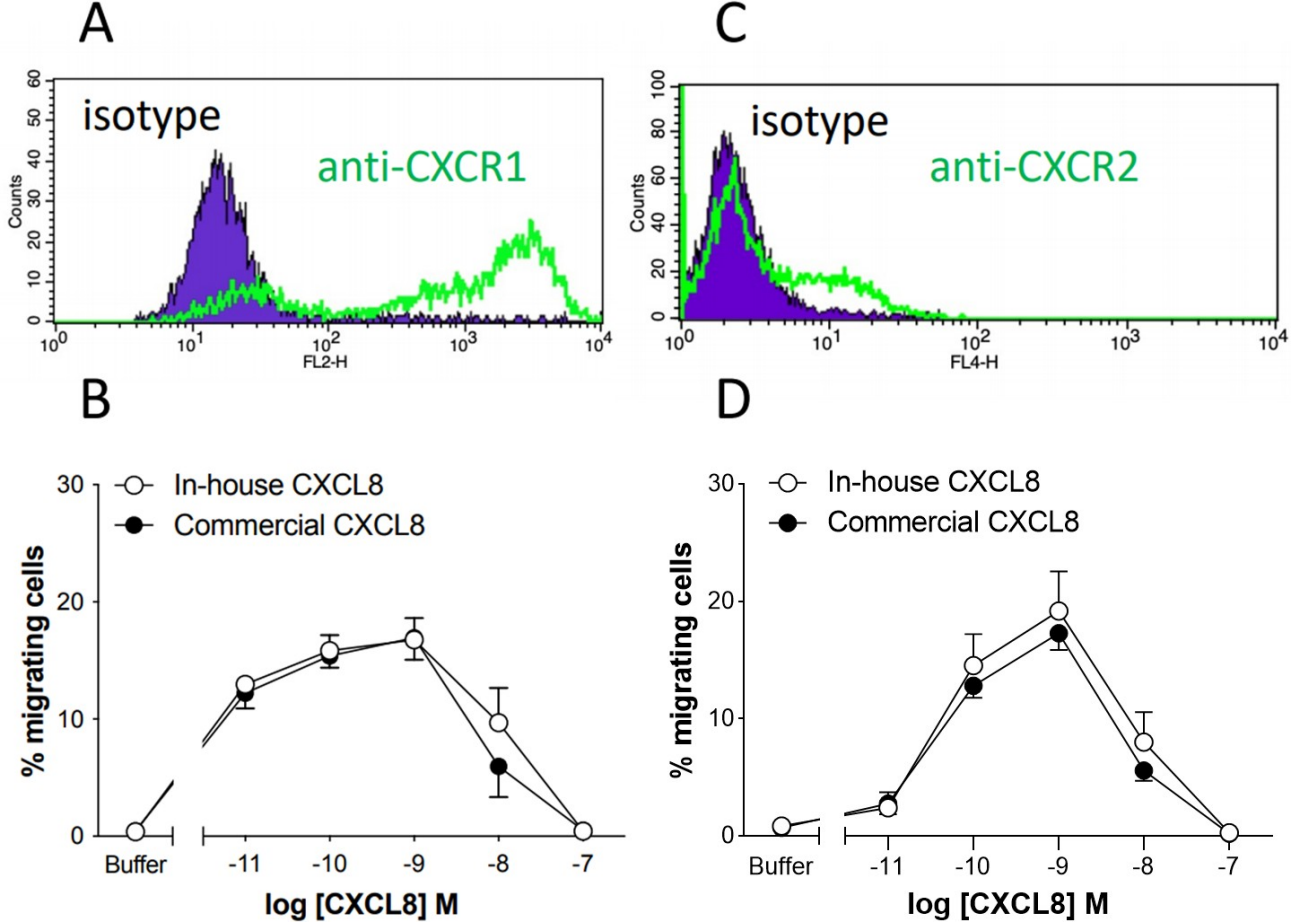
In-house produced CXCL8 is bioactive as deduced by chemotaxis assays. (A and C) Flow cytometry staining showing the cell surface expression of CXCR1 and CXCR2 on transfected L1.2 cells. Data are representative of three independent transfections. (B and D) The relative chemotactic activities of commercially available and in-house generated CXCL8 on CXCR1 and CXCR2 transfectants. Mean percentage migration is shown together with SEM (n = 3).

Plasmids encoding the human CXCL8 receptors CXCR1 and CXCR2 were introduced into the L1.2 cell line and following overnight treatment with sodium butyrate, cell surface expression of CXCR1 and CXCR2 was confirmed by flow cytometry (Fig 2A and 2C). CXCR1 and CXCR2 transfectants were then used to assess the bioactivity of CXCL8 using modified Boyden chamber chemotaxis assays. The precise concentration of our recombinant CXCL8 (6–77) produced in-house was determined using a CXCL8 ELISA. Serial dilutions of in-house and commercially available CXCL8 (6–77) (Bio-Techne) were used in the chemotaxis assays (Fig 2B and 2D). Bell-shaped chemotactic responses from both transfectants, typical of such assays were produced by both in-house and commercially available CXCL8. The efficacy of the responses from both CXCR1 and CXCR2 transfectant were similar, with approximately 20% of the transfected cells migrating at the optimal CXCL8 concentration. CXCL8 was found to induce more potent responses from CXCR1 transfectants than from CXCR2 transfectants, notably at the lowest concentrations of CXCL8 used. In both chemotaxis assays, the data from in-house CXCL8 and commercially available CXCL8 were indistinguishable.

### NMR analysis of CXCL8

NMR spectroscopy is a robust technique for assessing the folded state of proteins and has been used extensively to structurally characterise chemokines. One dimensional ^1^H spectrum was recorded to assess the folded state of CXCL8 produced in this study (S2 Fig). In aqueous buffer the spectrum yields well-dispersed amide and methyl regions indicative of a folded sample, which is consistent with published data of monomeric and dimeric forms [9, 37, 38].

**Fig 3:**
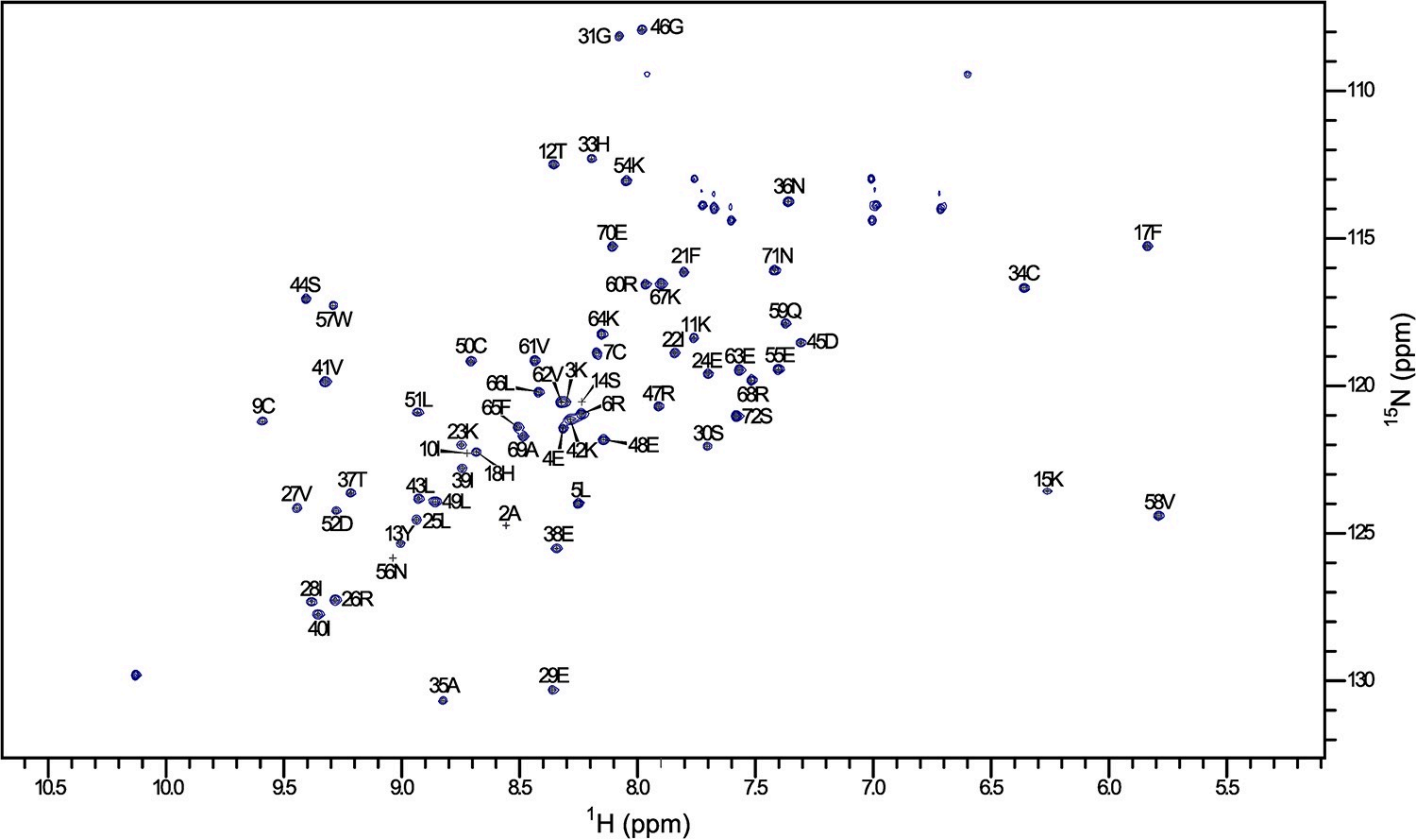
^1^H, ^15^N-HSQC spectrum of [*U*-^15^N]-labelled CXCL8 recorded at 298K. The spectrum is indicative of a folded sample with well dispersed and resolved peaks. 83 peaks are observed consistent with the expected number of backbone and side chain resonances. 67 peaks were assigned to the backbone yielding 99% sequence coverage, excluding the 4 proline residues. The N-terminal serine residue could not be assigned.

A (^1^H, ^15^N) HSQC spectrum of uniformly [*U*-^15^N]-labelled CXCL8 recorded at 0.5 mM is well-resolved, exhibits broad chemical shift dispersion typical of a native globular protein and is comparable to published spectra of folded CXCL8 (Fig 3) [9, 37, 38]. The spectrum exhibits 83 peaks in line with the expected number of backbone and side chain resonances for this 72 amino acid protein. X-ray crystallographic and NMR analysis have shown that CXCL8 is a homodimer at μM concentrations up to 2 mM, so it would be expected that a dimer is observed here [24, 25, 39]. To confirm this dimeric state, Size Exclusion Chromatography in tandem with Multi-Angle Laser Light Scattering (SEC-MALS) was carried out on CXCL8 at 0.5 mM. A single species was observed during the run, indicating sample homogeneity, with a molecular weight of 16.5 kDa ± 1.7% reported (S3 Fig). This is in line with the expected molecular weight of the dimeric form of CXCL8 produced in this study (16.7 kDa).

A ^1^H and ^15^N sequential assignment of CXCL8 was performed using ^15^N-HSQC-TOCSY and ^15^N-HSQC-NOESY spectra, which was comparable to published data [25, 40]. The assignment covers 99% of the backbone, excluding proline residues and the N-terminal serine residue which could not be assigned. A complete list of the ^1^H and ^15^N NMR chemical shifts assigned in this study are detailed in S1 Table.

The dimer interface of CXCL8 (6–77) was shown to be stabilised by hydrogen bonds between Strand 1, an anti-parallel β-sheet spanning residues 23 to 30, of each subunit [25]. Intermolecular NOEs were observable in our data, confirming this expected connectivity. We observed connectivity between 27HN-25HB2 and 25HB3, 29HN-24HA, 25HN-27HN, 25HN-27HB and 25HN-28HA, in line with the hydrogen bonding pattern elucidated by Clore et al (1990).

## Discussion

Here we present an efficient protocol for expressing soluble CXCL8 as a SUMO fusion in *E*. *coli* Shuffle T7 Express. We detail an effective purification schedule, encompassing nickel affinity purification, ULP1-mediated SUMO tag removal in a non-reducing environment and reverse IMAC purification separating untagged CXCL8 from the other tagged components. CXCL8 produced by this method is pure, correctly folded and bioactive, inducing chemotaxis of CXCR1 and CXCR2 transfectants with identical potency and efficacy to that commercially available CXCL8. Typically, 15 to 20 mg of CXCL8 are produced by this method per litre of cell culture.

The pET-32 Xa/LIC expression system, which requires Factor Xa for tag digestion, has been utilised extensively to produce CXCL8 [9–12]. Both ULP1 and Factor Xa have been employed to generate the correct N-terminus in recombinant protein production, which is a stringent requirement of functional chemokine production. However, Factor Xa mediated digestion often results in cleavage at undesired cryptic sites within the protein of interest, limiting the wider applicability of this system [13]. ULP1 is highly specific as it recognises the SUMO fold, a distinct advantage over Factor Xa, and can accommodate any residue at the N-terminus with the exception of proline [36]. Application of this expression and purification protocol to other chemokines could provide streamlined recombinant production of these proteins.

It should be noted that chemokines can also be produced via chemical synthesis routes, which carry considerable advantages over recombinant production, such as the ease of incorporating chemical modifications and the ability to generate multiple isoforms in one reaction schedule [41]. However, folding of the synthesised chemokine still requires optimisation and there is a requirement for expertise and equipment to run the synthesis cycle.

By producing soluble chemokines that are folded within the host, this methodology may have utility in the production of chemokines that diverge from the classic chemokine fold, such as those with an extra disulphide bond distal to the chemokine core. Structural determination of these recalcitrant proteins has shown them to be highly variable and has utilised refolding, in the case of CCL21 [42, 43], CCL23 [44] and CCL28 [45], or peptide synthesis routes, for CCL1 [46] and CCL15 [47]. However, challenging chemokines with an extra disulphide bond, such as CXCL16 and CXCL17, remain structurally uncharacterised. Refolding and synthetic routes have undoubtedly yielded results, but the method outlined here may simplify protein production at the levels required for structural characterisation while eliminating the requirement for the optimisation of protein refolding.

## Supporting information

S1 Raw images Image 1A is an uncropped gel image corresponding to Fig 1A. Image 1B is an uncropped gel image corresponding to—Fig 1B. Image S1 relates to S1 Fig. All lanes are marked in accordance with labelling in the manuscript and any unused lanes are marked with an X.
(TIF)

S1 Fig Soluble expression of CXCL8. SDS-PAGE analysis of CXCL8-SUMO3 expression in Shuffle T7 express *E*. *coli* cells in 10 ml M9 minimal media per condition. The lanes are labelled (1) uninduced control, (2) 0.1 mM IPTG at 16°C, (3) 0.5 mM IPTG at 16°C, (4) 1 mM IPTG at 16°C, (5) 0.1 mM IPTG at 30°C, (6) 0.5 mM IPTG at 30°C and (7) 1 mM IPTG at 30°C.
(TIF)

S2 Fig 1D 1H NMR spectrum of CXCL8. 1D ^1^H NMR spectra of CXCL8 were recorded at 100 μM. The spectrum exhibits well dispersed chemical shifts, indicative of a well-folded sample.
(TIF)

S3 Fig SEC-MALS of CXCL8 indicates dimerization. Differential refractive index and molar mass (g/mol) are plotted against elution volume. The differential refractive index is reported in blue. The molecular weight was calculated from the black line, which corresponds to the area of the peak used for this calculation. The reported molecular weight was 16.5 kDa ± 1.7%, in line with the expected molecular weight of the CXCL8 dimer which is 16.7 kDa.
(TIF)

S1 Table A complete list of the 1H and 15N NMR chemical shifts assigned in this study. (DOCX)
