# Supplementary figures and images for: A highly efficient method for the production and purification of recombinant human CXCL8
Source: PLoS One. 2021 Oct 15;16(10):e0258270. doi: 10.1371/journal.pone.0258270 (PMC8519433; doi:10.1371/journal.pone.0258270)

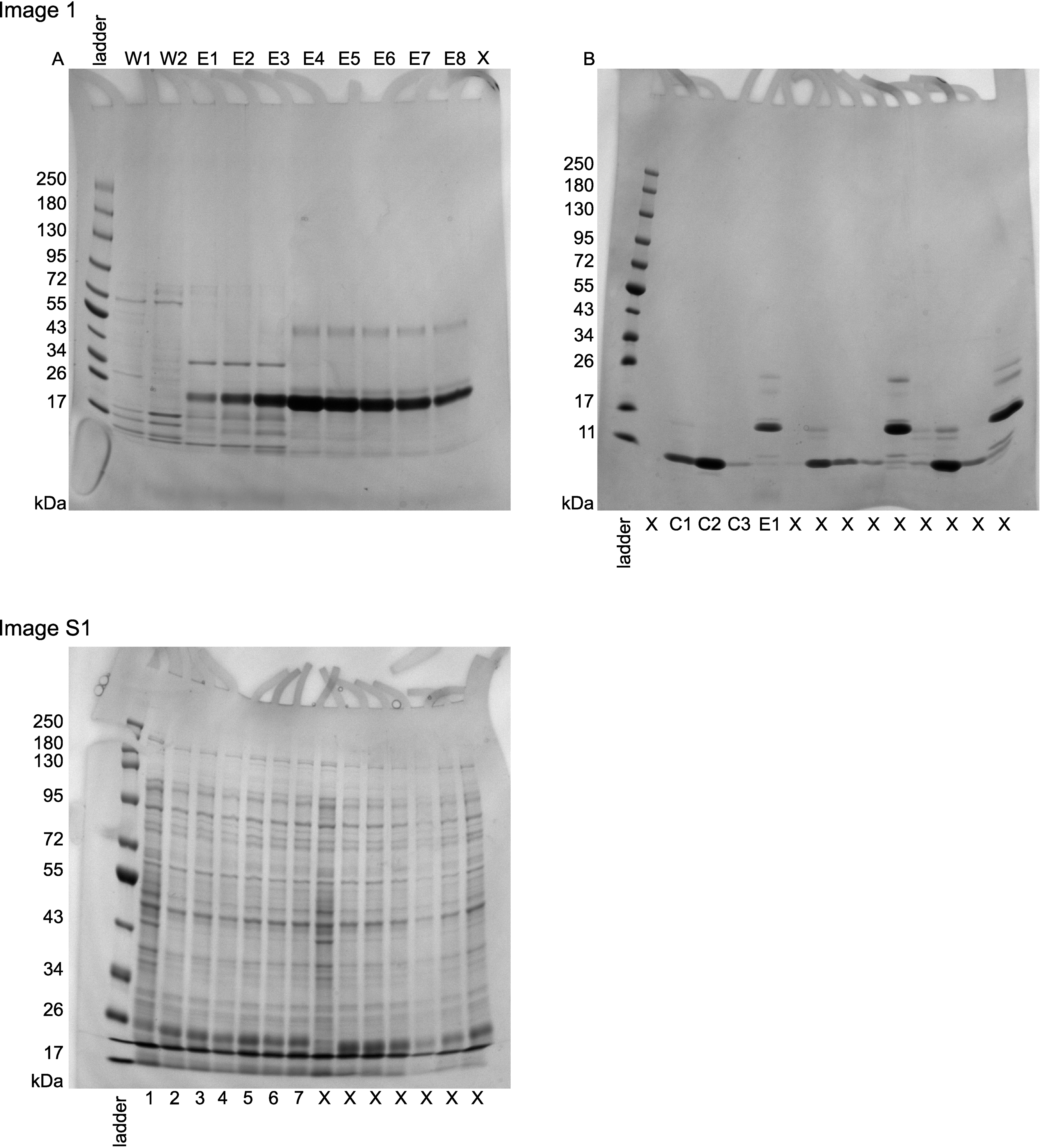

Supplement: S1 Raw images — Image 1B is an uncropped gel image corresponding to—Fig 1B. Image S1 relates to S1 Fig. All lanes are marked in accordance with labelling in the manuscript and any unused lanes are marked with an X. (TIF) [file pone.0258270.s001.tif]

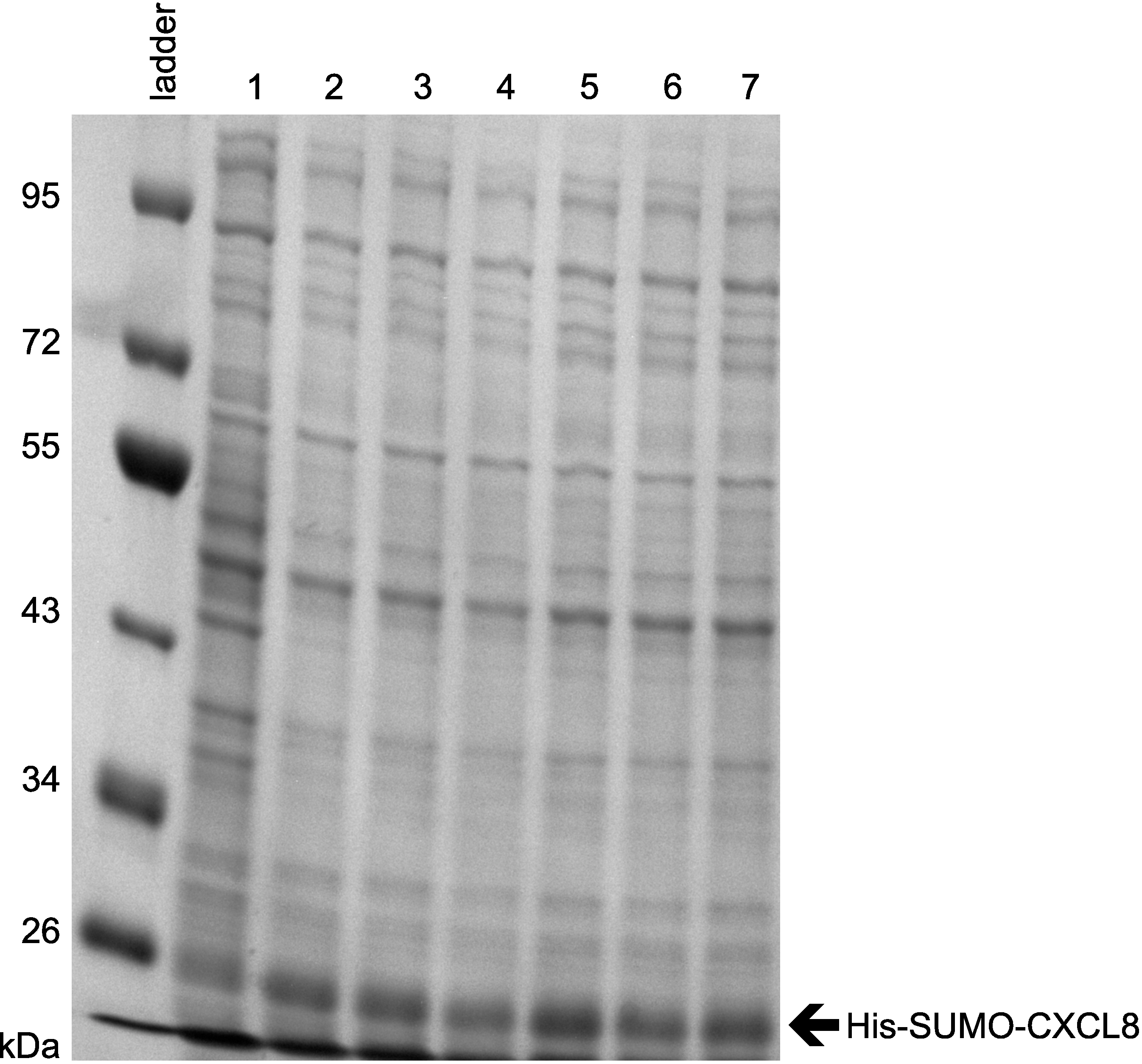

Supplement: S1 Fig — SDS-PAGE analysis of CXCL8-SUMO3 expression in Shuffle T7 express E. coli cells in 10 ml M9 minimal media per condition. The lanes are labelled (1) uninduced control, (2) 0.1 mM IPTG at 16°C, (3) 0.5 mM IPTG at 16°C, (4) 1 mM IPTG at 16°C, (5) 0.1 mM IPTG at 30°C, (6) 0.5 mM IPTG at 30°C and (7) 1 mM IPTG at 30°C. (TIF) [file pone.0258270.s002.tif]

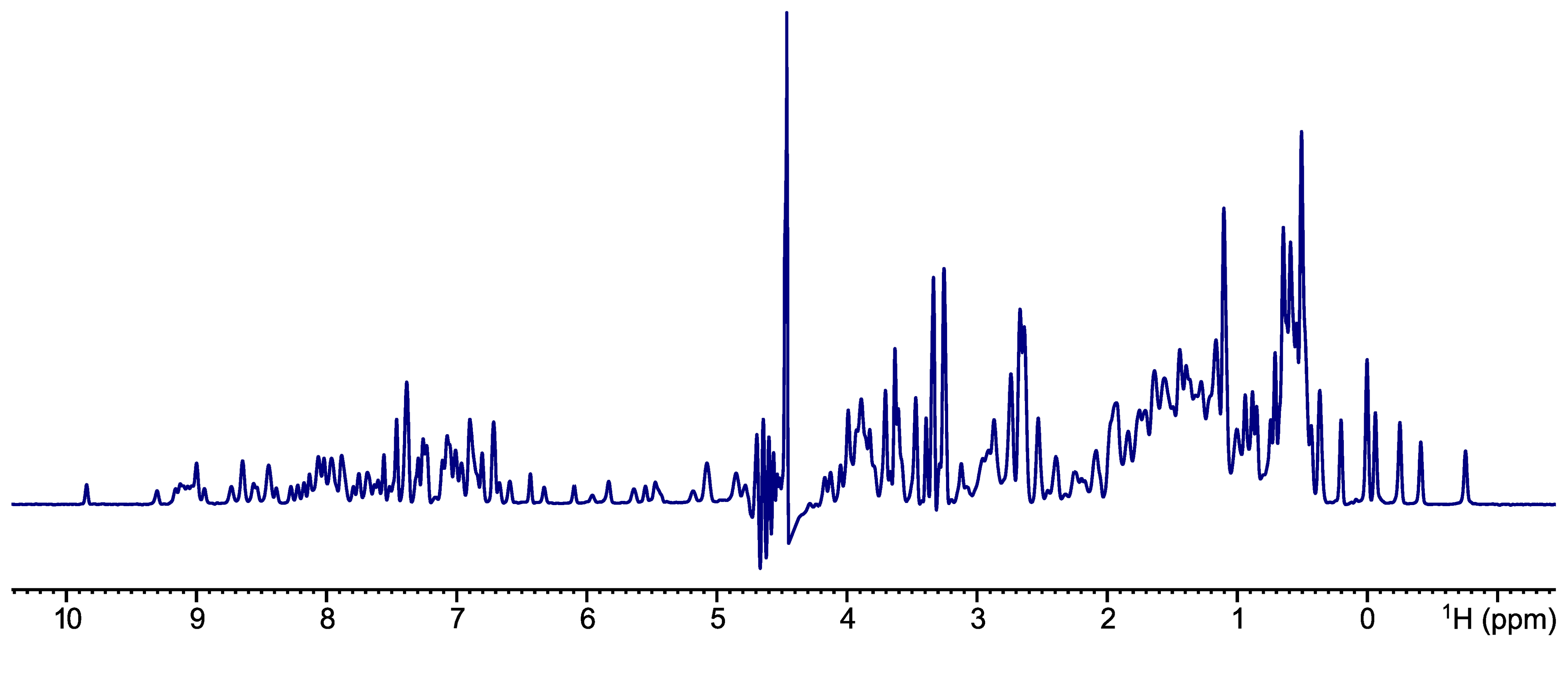

Supplement: S2 Fig — 1D 1H NMR spectra of CXCL8 were recorded at 100 μM. The spectrum exhibits well dispersed chemical shifts, indicative of a well-folded sample. (TIF) [file pone.0258270.s003.tif]

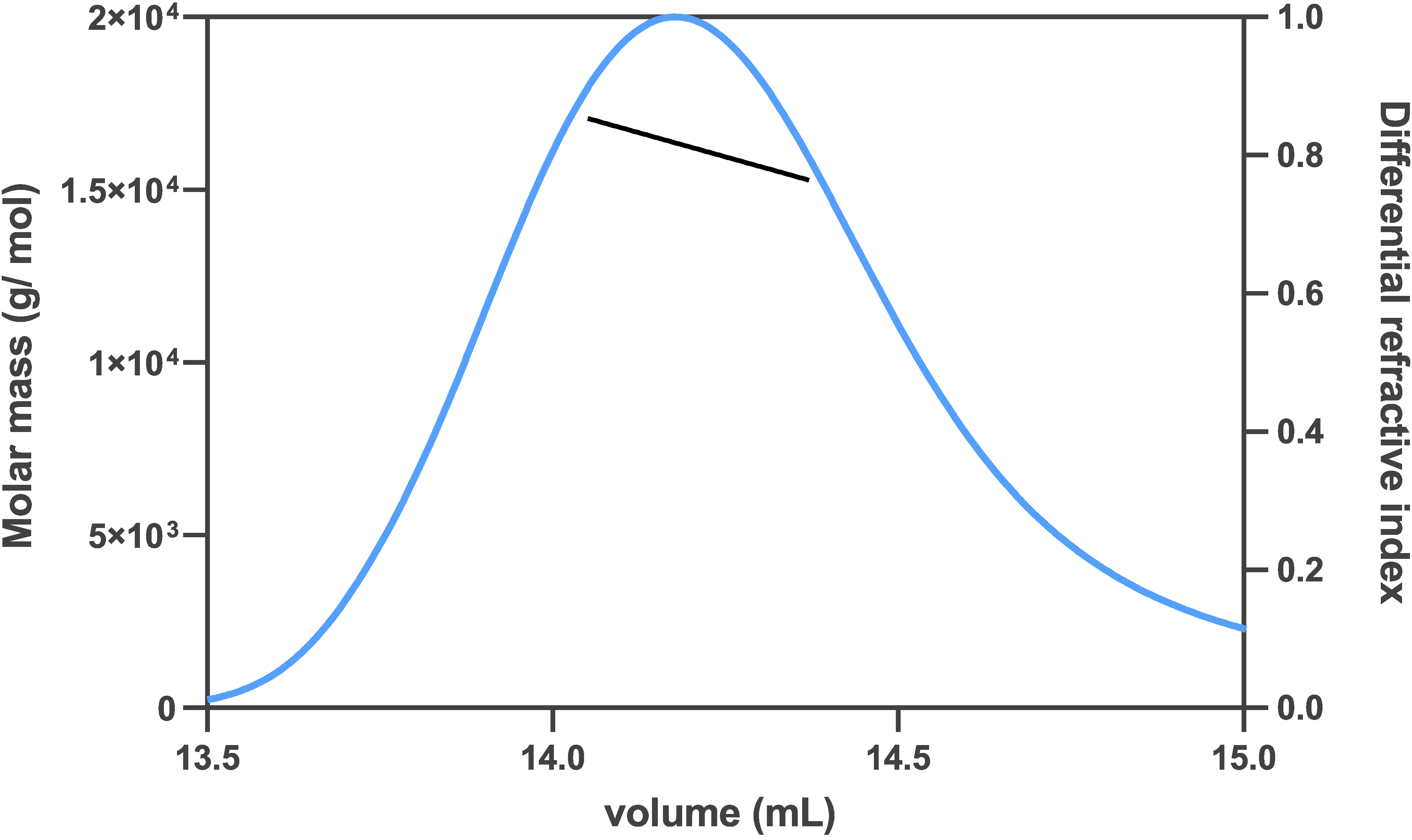

Supplement: S3 Fig — Differential refractive index and molar mass (g/mol) are plotted against elution volume. The differential refractive index is reported in blue. The molecular weight was calculated from the black line, which corresponds to the area of the peak used for this calculation. The reported molecular weight was 16.5 kDa ± 1.7%, in line with the expected molecular weight of the CXCL8 dimer which is 16.7 kDa. (TIF) [file pone.0258270.s004.tif]
