# Supplementary material for: A highly efficient method for the production and purification of recombinant human CXCL8
Source: PLoS One. 2021 Oct 15;16(10):e0258270. doi: 10.1371/journal.pone.0258270 (PMC8519433; doi:10.1371/journal.pone.0258270)
Supplement: S1 Table — (DOCX) [file pone.0258270.s005.docx]

| **Residue Number** | **Amino Acid of Residue** | **Atom Name** | **Shift (ppm)** | **Error (ppm)** |
| --- | --- | --- | --- | --- |
| 2 | Ala | H | 8.557 | 0 |
| 2 | Ala | N | 124.732 | 0 |
| 3 | Lys | H | 8.308 | 0.003 |
| 3 | Lys | HA | 4.215 | 0 |
| 3 | Lys | HB2 | 1.729 | 0 |
| 3 | Lys | HG2 | 1.412 | 0 |
| 3 | Lys | N | 120.554 | 0.012 |
| 4 | Glu | H | 8.318 | 0.001 |
| 4 | Glu | HA | 4.258 | 0.004 |
| 4 | Glu | HB2 | 1.933 | 0 |
| 4 | Glu | HG2 | 2.204 | 0 |
| 4 | Glu | N | 121.442 | 0.002 |
| 5 | Leu | H | 8.254 | 0.001 |
| 5 | Leu | HA | 4.416 | 0.002 |
| 5 | Leu | HB2 | 1.515 | 0 |
| 5 | Leu | HB3 | 1.708 | 0 |
| 5 | Leu | N | 123.979 | 0.007 |
| 6 | Arg | H | 8.259 | 0.012 |
| 6 | Arg | HA | 4.974 | 0.001 |
| 6 | Arg | HB2 | 1.792 | 0 |
| 6 | Arg | HB3 | 2.08 | 0 |
| 6 | Arg | HD2 | 3.193 | 0 |
| 6 | Arg | HG2 | 1.539 | 0 |
| 6 | Arg | N | 121.053 | 0.054 |
| 7 | Cys | H | 8.171 | 0.005 |
| 7 | Cys | HA | 4.667 | 0.006 |
| 7 | Cys | HB2 | 2.685 | 0.001 |
| 7 | Cys | HB3 | 4.07 | 0.001 |
| 7 | Cys | N | 118.944 | 0.053 |
| 8 | Gln | H | 12.22 | 0.005 |
| 8 | Gln | HA | 4.206 | 0 |
| 8 | Gln | HB2 | 1.998 | 0.002 |
| 8 | Gln | N | 126.805 | 0.008 |
| 9 | Cys | H | 9.591 | 0.001 |
| 9 | Cys | HA | 4.721 | 0 |
| 9 | Cys | HB2 | 2.69 | 0 |
| 9 | Cys | HB3 | 3.399 | 0 |
| 9 | Cys | N | 121.2 | 0.009 |
| 10 | Ile | H | 8.727 | 0.004 |
| 10 | Ile | HA | 4.152 | 0 |
| 10 | Ile | HG21 | 0.934 | 0 |
| 10 | Ile | HG22 | 0.934 | 0 |
| 10 | Ile | HG23 | 0.934 | 0 |
| 10 | Ile | N | 122.323 | 0.006 |
| 11 | Lys | H | 7.764 | 0.002 |
| 11 | Lys | HA | 4.687 | 0.001 |
| 11 | Lys | HB3 | 1.882 | 0 |
| 11 | Lys | HG2 | 1.416 | 0 |
| 11 | Lys | N | 118.374 | 0.011 |
| 12 | Thr | H | 8.358 | 0.002 |
| 12 | Thr | HA | 4.577 | 0.001 |
| 12 | Thr | HB | 4.174 | 0 |
| 12 | Thr | HG21 | 1.015 | 0 |
| 12 | Thr | HG22 | 1.015 | 0 |
| 12 | Thr | HG23 | 1.015 | 0 |
| 12 | Thr | N | 112.504 | 0.01 |
| 13 | Tyr | H | 9.007 | 0.001 |
| 13 | Tyr | HA | 4.694 | 0 |
| 13 | Tyr | HB2 | 3.155 | 0 |
| 13 | Tyr | N | 125.355 | 0.012 |
| 14 | Ser | H | 8.234 | 0.002 |
| 14 | Ser | HA | 4.83 | 0 |
| 14 | Ser | HB2 | 3.973 | 0 |
| 14 | Ser | N | 120.546 | 0 |
| 15 | Lys | H | 6.264 | 0.002 |
| 15 | Lys | HA | 4.475 | 0 |
| 15 | Lys | HB2 | 1.44 | 0 |
| 15 | Lys | HB3 | 1.589 | 0 |
| 15 | Lys | HG2 | 1.195 | 0 |
| 15 | Lys | N | 123.585 | 0.014 |
| 17 | Phe | H | 5.839 | 0.002 |
| 17 | Phe | HA | 4.945 | 0.005 |
| 17 | Phe | N | 115.27 | 0.004 |
| 18 | His | H | 8.685 | 0.001 |
| 18 | His | HA | 4.641 | 0 |
| 18 | His | N | 122.252 | 0.017 |
| 20 | Lys | H | 11.998 | 0.004 |
| 20 | Lys | HA | 4.312 | 0.002 |
| 20 | Lys | N | 126.784 | 0.006 |
| 21 | Phe | H | 7.807 | 0.002 |
| 21 | Phe | HA | 4.568 | 0.001 |
| 21 | Phe | HB2 | 2.949 | 0.001 |
| 21 | Phe | HB3 | 3.424 | 0.007 |
| 21 | Phe | N | 116.15 | 0.015 |
| 22 | Ile | H | 7.845 | 0.002 |
| 22 | Ile | HA | 3.666 | 0.001 |
| 22 | Ile | HB | 1.675 | 0 |
| 22 | Ile | HG13 | -0.106 | 0 |
| 22 | Ile | HG21 | -0.106 | 0 |
| 22 | Ile | HG22 | -0.106 | 0 |
| 22 | Ile | HG23 | -0.106 | 0 |
| 22 | Ile | N | 118.884 | 0.012 |
| 23 | Lys | H | 8.748 | 0.003 |
| 23 | Lys | HA | 4.992 | 0 |
| 23 | Lys | HB2 | 1.572 | 0 |
| 23 | Lys | HB3 | 1.97 | 0 |
| 23 | Lys | N | 121.985 | 0.028 |
| 24 | Glu | H | 7.706 | 0.003 |
| 24 | Glu | HA | 5.368 | 0.003 |
| 24 | Glu | HB2 | 1.954 | 0 |
| 24 | Glu | HB3 | 2.025 | 0 |
| 24 | Glu | HG2 | 2.238 | 0 |
| 24 | Glu | N | 119.564 | 0.012 |
| 25 | Leu | H | 8.941 | 0.002 |
| 25 | Leu | HA | 4.929 | 0.004 |
| 25 | Leu | HB2 | 1.303 | 0.007 |
| 25 | Leu | HB3 | 1.865 | 0.001 |
| 25 | Leu | N | 124.515 | 0.034 |
| 26 | Arg | H | 9.283 | 0.003 |
| 26 | Arg | HA | 5.736 | 0 |
| 26 | Arg | N | 127.257 | 0.005 |
| 27 | Val | H | 9.448 | 0.002 |
| 27 | Val | HA | 5.132 | 0 |
| 27 | Val | HB | 2.235 | 0 |
| 27 | Val | HG11 | 0.765 | 0 |
| 27 | Val | HG12 | 0.765 | 0 |
| 27 | Val | HG13 | 0.765 | 0 |
| 27 | Val | HG21 | 0.992 | 0 |
| 27 | Val | HG22 | 0.992 | 0 |
| 27 | Val | HG23 | 0.992 | 0 |
| 27 | Val | N | 124.122 | 0.023 |
| 28 | Ile | H | 9.387 | 0.003 |
| 28 | Ile | HA | 4.655 | 0.009 |
| 28 | Ile | HB | 2.07 | 0 |
| 28 | Ile | N | 127.315 | 0.003 |
| 29 | Glu | H | 8.359 | 0.007 |
| 29 | Glu | HA | 4.463 | 0 |
| 29 | Glu | HB3 | 2.472 | 0 |
| 29 | Glu | N | 130.318 | 0.008 |
| 30 | Ser | H | 7.709 | 0.003 |
| 30 | Ser | HA | 4.174 | 0.001 |
| 30 | Ser | HB2 | 3.757 | 0 |
| 30 | Ser | HB3 | 3.866 | 0.001 |
| 30 | Ser | N | 122.062 | 0.017 |
| 31 | Gly | H | 8.08 | 0 |
| 31 | Gly | HA2 | 4.091 | 0 |
| 31 | Gly | HA3 | 4.254 | 0 |
| 31 | Gly | N | 108.126 | 0.022 |
| 33 | His | H | 8.193 | 0.003 |
| 33 | His | HA | 4.26 | 0.004 |
| 33 | His | HB2 | 2.935 | 0 |
| 33 | His | N | 112.304 | 0.003 |
| 34 | Cys | H | 6.358 | 0.003 |
| 34 | Cys | HA | 4.462 | 0.004 |
| 34 | Cys | HB2 | 2.592 | 0 |
| 34 | Cys | HB3 | 2.992 | 0.006 |
| 34 | Cys | N | 116.69 | 0.005 |
| 35 | Ala | H | 8.829 | 0.006 |
| 35 | Ala | HA | 4.139 | 0.006 |
| 35 | Ala | HB1 | 1.374 | 0 |
| 35 | Ala | HB2 | 1.374 | 0 |
| 35 | Ala | HB3 | 1.374 | 0 |
| 35 | Ala | N | 130.691 | 0.007 |
| 36 | Asn | H | 7.362 | 0.001 |
| 36 | Asn | HA | 5.129 | 0 |
| 36 | Asn | HB2 | 2.542 | 0 |
| 36 | Asn | HB3 | 2.8 | 0 |
| 36 | Asn | N | 113.759 | 0.008 |
| 37 | Thr | H | 9.221 | 0.004 |
| 37 | Thr | HA | 4.518 | 0.002 |
| 37 | Thr | HB | 4.097 | 0 |
| 37 | Thr | HG21 | 0.924 | 0 |
| 37 | Thr | HG22 | 0.924 | 0 |
| 37 | Thr | HG23 | 0.924 | 0 |
| 37 | Thr | N | 123.636 | 0.003 |
| 38 | Glu | H | 8.342 | 0.002 |
| 38 | Glu | HA | 4.971 | 0.002 |
| 38 | Glu | HB2 | 2.003 | 0 |
| 38 | Glu | HB3 | 1.887 | 0 |
| 38 | Glu | HG2 | 2.439 | 0 |
| 38 | Glu | N | 125.504 | 0.019 |
| 39 | Ile | H | 8.748 | 0.005 |
| 39 | Ile | HA | 4.871 | 0.002 |
| 39 | Ile | HB | 1.936 | 0 |
| 39 | Ile | HG21 | 0.66 | 0 |
| 39 | Ile | HG22 | 0.66 | 0 |
| 39 | Ile | HG23 | 0.66 | 0 |
| 39 | Ile | N | 122.813 | 0.018 |
| 40 | Ile | H | 9.358 | 0.002 |
| 40 | Ile | HA | 5.071 | 0 |
| 40 | Ile | HB | 1.573 | 0 |
| 40 | Ile | HG21 | 0.767 | 0 |
| 40 | Ile | HG22 | 0.767 | 0 |
| 40 | Ile | HG23 | 0.767 | 0 |
| 40 | Ile | N | 127.746 | 0.01 |
| 41 | Val | H | 9.327 | 0.002 |
| 41 | Val | HA | 5.379 | 0.001 |
| 41 | Val | N | 119.876 | 0.011 |
| 42 | Lys | H | 8.287 | 0.002 |
| 42 | Lys | HA | 5.151 | 0.002 |
| 42 | Lys | HB3 | 1.74 | 0 |
| 42 | Lys | HG2 | 1.301 | 0 |
| 42 | Lys | N | 121.15 | 0.011 |
| 43 | Leu | H | 8.932 | 0.002 |
| 43 | Leu | HA | 5.474 | 0.001 |
| 43 | Leu | HB2 | 1.428 | 0 |
| 43 | Leu | HB3 | 2.093 | 0 |
| 43 | Leu | N | 123.83 | 0.004 |
| 44 | Ser | H | 9.407 | 0.003 |
| 44 | Ser | HA | 4.111 | 0 |
| 44 | Ser | N | 117.057 | 0.008 |
| 45 | Asp | H | 7.31 | 0.001 |
| 45 | Asp | HA | 4.573 | 0 |
| 45 | Asp | HB2 | 2.47 | 0 |
| 45 | Asp | HB3 | 3.047 | 0 |
| 45 | Asp | N | 118.535 | 0.012 |
| 46 | Gly | H | 7.981 | 0.003 |
| 46 | Gly | HA2 | 3.539 | 0.005 |
| 46 | Gly | HA3 | 4.356 | 0.001 |
| 46 | Gly | N | 107.911 | 0.01 |
| 47 | Arg | H | 7.915 | 0.003 |
| 47 | Arg | HA | 4.067 | 0.001 |
| 47 | Arg | HB2 | 1.827 | 0 |
| 47 | Arg | HB3 | 1.813 | 0 |
| 47 | Arg | HG2 | 1.527 | 0 |
| 47 | Arg | HG3 | 1.472 | 0 |
| 47 | Arg | N | 120.712 | 0.007 |
| 48 | Glu | H | 8.147 | 0.002 |
| 48 | Glu | HA | 5.353 | 0 |
| 48 | Glu | HB3 | 1.874 | 0 |
| 48 | Glu | HG2 | 2.035 | 0 |
| 48 | Glu | N | 121.836 | 0.008 |
| 49 | Leu | H | 8.859 | 0.001 |
| 49 | Leu | HA | 4.734 | 0 |
| 49 | Leu | HB2 | 1.363 | 0 |
| 49 | Leu | N | 123.935 | 0.006 |
| 50 | Cys | H | 8.709 | 0.001 |
| 50 | Cys | HA | 5.936 | 0.001 |
| 50 | Cys | HB2 | 3.171 | 0 |
| 50 | Cys | HB3 | 4.109 | 0 |
| 50 | Cys | N | 119.153 | 0.008 |
| 51 | Leu | H | 8.935 | 0.002 |
| 51 | Leu | HA | 4.935 | 0.003 |
| 51 | Leu | HB2 | 1.22 | 0.003 |
| 51 | Leu | N | 120.913 | 0.006 |
| 52 | Asp | H | 9.278 | 0 |
| 52 | Asp | HB2 | 2.533 | 0 |
| 52 | Asp | HB3 | 2.948 | 0 |
| 52 | Asp | N | 124.231 | 0.005 |
| 54 | Lys | H | 8.052 | 0.002 |
| 54 | Lys | HA | 4.014 | 0 |
| 54 | Lys | HB2 | 1.837 | 0 |
| 54 | Lys | HG2 | 1.444 | 0 |
| 54 | Lys | N | 113.059 | 0.018 |
| 55 | Glu | H | 7.406 | 0.002 |
| 55 | Glu | HA | 4.221 | 0 |
| 55 | Glu | HB2 | 1.72 | 0 |
| 55 | Glu | HG2 | 2.154 | 0 |
| 55 | Glu | N | 119.439 | 0.01 |
| 56 | Asn | H | 9.039 | 0.001 |
| 56 | Asn | HA | 4.115 | 0.002 |
| 56 | Asn | HB2 | 2.809 | 0 |
| 56 | Asn | HB3 | 2.954 | 0 |
| 56 | Asn | N | 125.844 | 0.011 |
| 57 | Trp | H | 9.291 | 0.001 |
| 57 | Trp | HA | 4.267 | 0 |
| 57 | Trp | HB2 | 3.25 | 0 |
| 57 | Trp | HB3 | 3.499 | 0 |
| 57 | Trp | HE1 | 10.13 | 0 |
| 57 | Trp | N | 117.279 | 0.008 |
| 57 | Trp | NE1 | 129.809 | 0 |
| 58 | Val | H | 5.792 | 0.003 |
| 58 | Val | HA | 2.678 | 0 |
| 58 | Val | HB | 1.905 | 0 |
| 58 | Val | N | 124.423 | 0.014 |
| 59 | Gln | H | 7.372 | 0.004 |
| 59 | Gln | HA | 3.592 | 0 |
| 59 | Gln | HB2 | 2.029 | 0 |
| 59 | Gln | N | 117.881 | 0.025 |
| 60 | Arg | H | 7.968 | 0.002 |
| 60 | Arg | HA | 4.204 | 0 |
| 60 | Arg | HB2 | 1.904 | 0 |
| 60 | Arg | HB3 | 1.985 | 0 |
| 60 | Arg | N | 116.564 | 0.007 |
| 61 | Val | H | 8.436 | 0.006 |
| 61 | Val | HA | 3.914 | 0 |
| 61 | Val | N | 119.142 | 0.013 |
| 62 | Val | H | 8.328 | 0.003 |
| 62 | Val | HA | 3.557 | 0 |
| 62 | Val | HB | 2.136 | 0 |
| 62 | Val | HG11 | 0.917 | 0 |
| 62 | Val | HG12 | 0.917 | 0 |
| 62 | Val | HG13 | 0.917 | 0 |
| 62 | Val | N | 120.586 | 0.021 |
| 63 | Glu | H | 7.57 | 0.003 |
| 63 | Glu | HA | 3.901 | 0 |
| 63 | Glu | HB2 | 2.181 | 0 |
| 63 | Glu | N | 119.469 | 0.005 |
| 64 | Lys | H | 8.155 | 0.002 |
| 64 | Lys | HA | 3.986 | 0 |
| 64 | Lys | HB2 | 1.945 | 0 |
| 64 | Lys | N | 118.244 | 0.017 |
| 65 | Phe | H | 8.511 | 0.001 |
| 65 | Phe | HA | 4.213 | 0.002 |
| 65 | Phe | HB2 | 3.209 | 0 |
| 65 | Phe | N | 121.409 | 0.015 |
| 66 | Leu | H | 8.422 | 0.003 |
| 66 | Leu | HA | 3.43 | 0.009 |
| 66 | Leu | N | 120.231 | 0.006 |
| 67 | Lys | H | 7.904 | 0.002 |
| 67 | Lys | HA | 3.901 | 0 |
| 67 | Lys | N | 116.554 | 0.025 |
| 68 | Arg | H | 7.518 | 0.001 |
| 68 | Arg | HA | 4.013 | 0 |
| 68 | Arg | HB2 | 1.935 | 0 |
| 68 | Arg | N | 119.815 | 0.014 |
| 69 | Ala | H | 8.483 | 0.01 |
| 69 | Ala | HA | 3.962 | 0 |
| 69 | Ala | HB1 | 0.819 | 0 |
| 69 | Ala | HB2 | 0.819 | 0 |
| 69 | Ala | HB3 | 0.819 | 0 |
| 69 | Ala | N | 121.72 | 0.007 |
| 70 | Glu | H | 8.11 | 0.008 |
| 70 | Glu | HA | 3.804 | 0 |
| 70 | Glu | HB2 | 2.05 | 0 |
| 70 | Glu | N | 115.288 | 0.048 |
| 71 | Asn | H | 7.42 | 0 |
| 71 | Asn | HA | 4.757 | 0 |
| 71 | Asn | HB2 | 2.681 | 0 |
| 71 | Asn | HB3 | 2.946 | 0.004 |
| 71 | Asn | N | 116.086 | 0.003 |
| 72 | Ser | H | 7.583 | 0.002 |
| 72 | Ser | HA | 4.28 | 0 |
| 72 | Ser | HB2 | 3.887 | 0 |
| 72 | Ser | N | 121.045 | 0.014 |
